# Coaching While Waiting for Autism Spectrum Disorder Assessment: A Pilot Feasibility Study for a Randomized Controlled Trial on Occupational Performance Coaching and Service Navigation

**DOI:** 10.1007/s10803-022-05558-3

**Published:** 2022-04-30

**Authors:** Charmaine Bernie, Katrina Williams, Fiona Graham, Tamara May

**Affiliations:** 1 Department of Paediatrics, The University of Melbourne, Melbourne, Australia; 2 Department of Allied Health, The Royal Children’s Hospital, Melbourne, Australia; 3 Southern Cross University, Gold Coast, Australia; 4 Murdoch Children’s Research Institute, Melbourne, Victoria Australia; 5 Developmental Paediatrics, Monash Children’s Hospital, Melbourne, Australia; 6 Department of Paediatrics, Monash University, Clayton, Australia; 7 Rehabilitation Teaching and Research Unit, Department of Medicine, University of Otago, Wellington, New Zealand

**Keywords:** Coaching, Child, Parent, Waiting list, Autism spectrum disorder, Feasibility

## Abstract

**Aim:**

To determine whether short-phase Occupational Performance Coaching combined with service navigation support is feasible for families waiting for autism assessment.

**Method:**

A pilot feasibility study was conducted using a blinded randomization procedure that allocated participants to one of three trial arms: (1) face-to-face coaching, (2) videoconference coaching, and (3) usual care. Outcomes included a retention aim of 70–80%, goal attainment and secondary standardised measures of adaptive behaviour, social skills, parenting stress, service access and family quality of life.

**Results:**

Caregivers and children (n = 16, child mean age of 3 years 7 months) were recruited following referral for an autism assessment. Retention was 75%, with change scores in performance and satisfaction of selected goals higher in the intervention groups than the usual care group.

**Interpretation:**

Findings support progression to a future randomized controlled trial assessing intervention efficacy.

Between 1 and 4% of Australian children are diagnosed with an autism spectrum disorder, herein referred to as autism (May et al., 2017). With onset in early childhood, the disorder is characterised by impairments in social communication and behaviour (American-Psychiatric-Association, 2013). Paediatricians, psychiatrists, and allied health professionals including psychologists, speech pathologists and occupational therapists can be sought to assess a child, working individually or in multidisciplinary teams (Whitehouse et al., 2018; Williams et al., 2014). In public health settings, there is a shortage of assessment services when autism-related concerns exist (Bent et al., 2015; Randall, Albein-Urios, Brignell, Gulenc, Hennel, Coates, Symeonides, Hiscock, Marraffa, Silove, Bayl, Woolfenden, & Williams, 2016).

Debate continues nationally and internationally between the notions of comprehensive autism assessment practices, and the need for early diagnoses to support appropriate service access (McKenzie, Forsyth, O’Hare, McClure, Rutherford, Murray, & Irvine, 2016). As a result, children and families can have unique and lengthy pathways to autism assessment (Hennel, Coates, Symeonides, Gulenc, Smith, Price, & Hiscock, 2016; Ward et al., 2016). The delays and confusion along the path from recognition of concerns to assessment can add to stress and concern for parents and families, and delay service access that could assist with each child’s immediate and long-term development Batool & Khurshid, 2015; Gibbs et al., 2019; Rivard et al., 2014; Whitehouse, Varcin, Alvares, Barbaro, Bent, Boutrus, Chetcuti, Cooper, Clark, & Davidson, 2019; Whitehouse 2017).

National guidelines in Australia have sought to address these issues by recommending standardised diagnostic practices (Whitehouse et al., 2018). Functional assessment and intervention are suggested to occur prior to, or concurrently with, the diagnostic assessment process. These guidelines remain broad to ensure their fit with a wide range of settings across Australia. There remains a lack of clarity around best-practice functional assessment and guidance in the pre-diagnostic stage, and lost opportunities continue to be lamented by researchers in the field (Gibbs et al., 2019; Vivanti & Volkmar, 2019).

Function-focused and goal-directed coaching strategies, such as Occupational Performance Coaching (OPC), are gaining support as an evidence-based intervention for parents of children with neurodevelopmental difficulties (Graham et al., 2013; Rivard et al., 2016; Schwellnus et al., 2020; Ward et al., 2019). OPC is a function-focused coaching approach where participants are supported to develop and implement strategies in the context of self-identified participatory goals (Graham et al., 2020). The approach has been used with a variety of populations, including parents of children with disabilities (Angelin et al., 2021; Graham et al., 2020; Kessler et al., 2017). Goal-directed coaching has been delivered via face-to-face (f2f) and telehealth or videoconference modalities with reported success (Boisvert et al., 2010; Little et al., 2018; Ward et al., 2019). Telehealth or videoconference-delivered interventions, have become increasingly favoured and appropriately prioritised in research following the COVID-19 pandemic (Eapen et al., 2021). These are now deemed to be a critical modality for supporting access to interventions, regardless of rural or metropolitan geographical locations (Camden & Silva, 2021; Eapen et al., 2021).

Service navigation and parent support group strategies have been trialled previously with parents of children waiting for autism assessment, with positive outcomes reported relating to assessment completion and parent knowledge (Connolly & Gersch, 2013; Feinberg, Abufhele, Sandler, Augustyn, Cabral, Chen, Diaz Linhart, Cesar Levesque, Aebi, & Silverstein, 2016).

As yet, a brief, individualised coaching intervention combined with service navigation support has not been trialled in a three-arm study, inclusive of a telehealth arm, with parents of children on an autism diagnostic waitlist. Our pilot and feasibility trial aimed to:

1. assess the feasibility, including constructs of acceptability, practicality, and preliminary efficacy, of an RCT study design exploring coaching and service navigation support for families of children waiting for ASD assessment.
2. inform protocol planning for a future RCT to assess efficacy of coaching and service navigation support via face-to-face and videoconference modalities.

## Methods

### Participants

Participants were identified following referral to either The Royal Children’s Hospital Melbourne (RCH) or Djerriwarrh Health Services in Melton (herein referred to as Melton) for an autism assessment, from December 2018 to June 2019. For referrals relating to children aged 0–7 years (n = 185), parents were sent study information along with their referral outcome letter, and invited to contact the investigator for more information. A follow-up phone call was permitted for the RCH clinic if families did not respond. Interested participants were excluded if the child had already been scheduled for accessed an autism assessment (n = 3), or a parent was currently engaged in regular coaching or mental health support sessions (n = 2). Eligible families who provided written consent participated in baseline measure completion, before being allocated to one of 3 study arms, as below. Enrolled participants were reimbursed for travel and/or parking costs where and when these were incurred, in line with ethics approval from RCH Human Research Ethics Committee.

### Intervention

Participants were randomly allocated to one of three study arms; usual-care, videoconference coaching or face-to-face (f2f) coaching. Usual care consisted of existing service access outside of the assessment service to which the child was referred, and telephone access to a part-time assessment service coordinator as required. Participants in the intervention arms received 4 sessions of a manualised intervention, OPC, which has previously been described in detail (Bernie et al., 2021; Graham et al., 2020). In brief, OPC is an evidence-based, function-focused coaching approach, built upon principles of connection, sharing and structure. The approach supports goal attainment for individuals and families through a focus on occupation and enablement. Four sessions of OPC were provided in addition to direct, on-demand service navigation support available to all participants, either face to face or via videoconference modality. Further intervention details are available in the study protocol.

There were no major deviations from the published protocol. Two study participants in the intervention arms accessed an autism assessment at the RCH prior to the 4th session of coaching. The final session of coaching in this instance included a focus on intervention conclusion, and discussing any impact the diagnostic outcome may have had on goal-related strategies or directions developed in previous sessions.

### Primary Outcome Measures

Recruitment and retention were calculated at recruitment close and study conclusion. Other primary outcome measures, including goal attainment, were collected at baseline (T_0_), and follow-up (T_1_). For families with two parents participating, measures were completed via consensus coding.

Measures were mapped onto feasibility constructs as outlined in the study protocol (Bernie et al., 2021), which included acceptability, practicality, demand, adaptability, and preliminary efficacy. Demand was measured through recruitment, with an aim of between 18 and 24 participants, acceptability and progression to a future RCT via a retention goal of 70–80%, and a post-intervention questionnaire, and preliminary efficacy through functional goal attainment measured by the Canadian Occupational Performance Measure (COPM), 6th Edition (Law et al., 2017). The COPM was administered at baseline (T_0_) and follow-up (T_1_), with the support of an interpreter for participants who were not proficient in English. When completing the COPM at baseline, parents identified areas of concern in daily occupations and then prioritised them. These could be concerns for their child or family relating to work or school, self-care activities, leisure or play at home or in the community. Areas of concern rated by the parent(s) for current performance and satisfaction, with higher scores representing higher performance and satisfaction levels.

### Secondary Outcome Measures

Participant completion rates and measurement at T_0_ and T_1_ were completed, including the Vinelands Adaptive Behaviour Scales (VABS) 3 (Sparrow et al., 2016), the Social Responsiveness Scale (SRS) 2 (Constantino & Gruber, 2012), the Beech Family Quality of Life Scale (FQOL) (Park, Hoffman, Marquis, Turnbull, Poston, Mannan, Wang, & Nelson, 2003), and the short form of the Parenting Stress Index (PSI) (Abidin, 1990). Baseline information including demographic data such as postcode and current service access was also collected via parent questionnaire and included direct reporting of medical and allied health professionals being accessed at baseline and follow-up. Socioeconomic status (Australian Bureau of Statistics, 2011) and other related data are presented in Table 1.

**Table 1:** Retained versus Withdrawn Participants (N = 16)

Variable	Arm A Usual-Care	Arm B Video-conference Coaching	Arm C Face-to-face Coaching	Completed Total	Withdrawn
N (%)	4 (25)	5 (31)	3 (19)	12 (75)	4 (25)
Gender N, M:F	0:4	4:1	3:0	7:5	4:0
Child age mean as years, months (SD)	3,6 (1.2)	3,8 (1.3)	3,7 (1.2)	3,7 (1.1)	4,3 (0.2)
Child has older sibling/s n (%)	3 (75)	3 (60)	2 (67)	8 (66)	1 (25)
Sibling with autism n (%)	0 (0)	1 (20)	1 (33)	2 (17)	0 (0)
Birth Mother main respondent n (%)	4 (100)	5 (100)	3 (100)	12 (100)	3 (75)
Both parents participants n (%)	0 (0)	1 (20)	2 (67)	3 (25)	0 (0)
Single parent n (%)	1 (25)	1 (20)	0 (0)	2 (17)	1 (25)
Parent education level diploma or above n (%)	3 (75)	4 (80)	1 (33)	8 (66)	2 (50)
Child in educational setting n (%)	4 (100)	4 (80)	2 (67)	10 (83)	3 (75)
Services at baseline mean (SD)	1.75 (1.71)	1.60 (1.52)	1.33 (0.58)	1.58 (1.31)	2.33 (0.58)
SES Decile mean (SD)	5.5 (2.4)	7.6 (2.9)	7.3 (1.5)	6.8 (2.4)	3.5 (1.9)
General Practitioner referred n (%)	4 (100)	3 (60)	0 (0)	7 (58)	2 (50)
Paediatrician referred n (%)	0 (0)	1 (20)	3 (100)	4 (33)	1 (25)
Interpreter required n (%)	0 (0)	1 (20)	1 (33)	2 (17)	0 (0)

### Implementation and Practicality

The Measure of Processes of Care − 20 (MPOC-20) (King et al., 2004) was used for parent ratings about care from current services, and to assess feasibility constructs of implementation and practicality. Parents reported on the nature and family-centredness of care received by rating provisions of specific and general information, respectful and coordinated care, and enabling and partnership on a scale, from “not at all” or 1 to “a very great extent” or 7. For the purposes of this study, parents were asked to provide one aggregate rating for all current services received, including receipt of the study intervention for participants in intervention arms at follow-up (T_1_).

### Statistical Analysis

Analyses included proportions for recruitment and retention (expected 70–80%), and change scores in COPM “performance” and “satisfaction” ratings, with a change of 2 points considered to be clinically significant (Verkerk et al., 2006). The direction of change across a number of secondary outcomes measuring child adaptive skills, social skills, service access, parenting stress and family quality of life were also explored (detailed below).

## Results

**Fig. 1:**
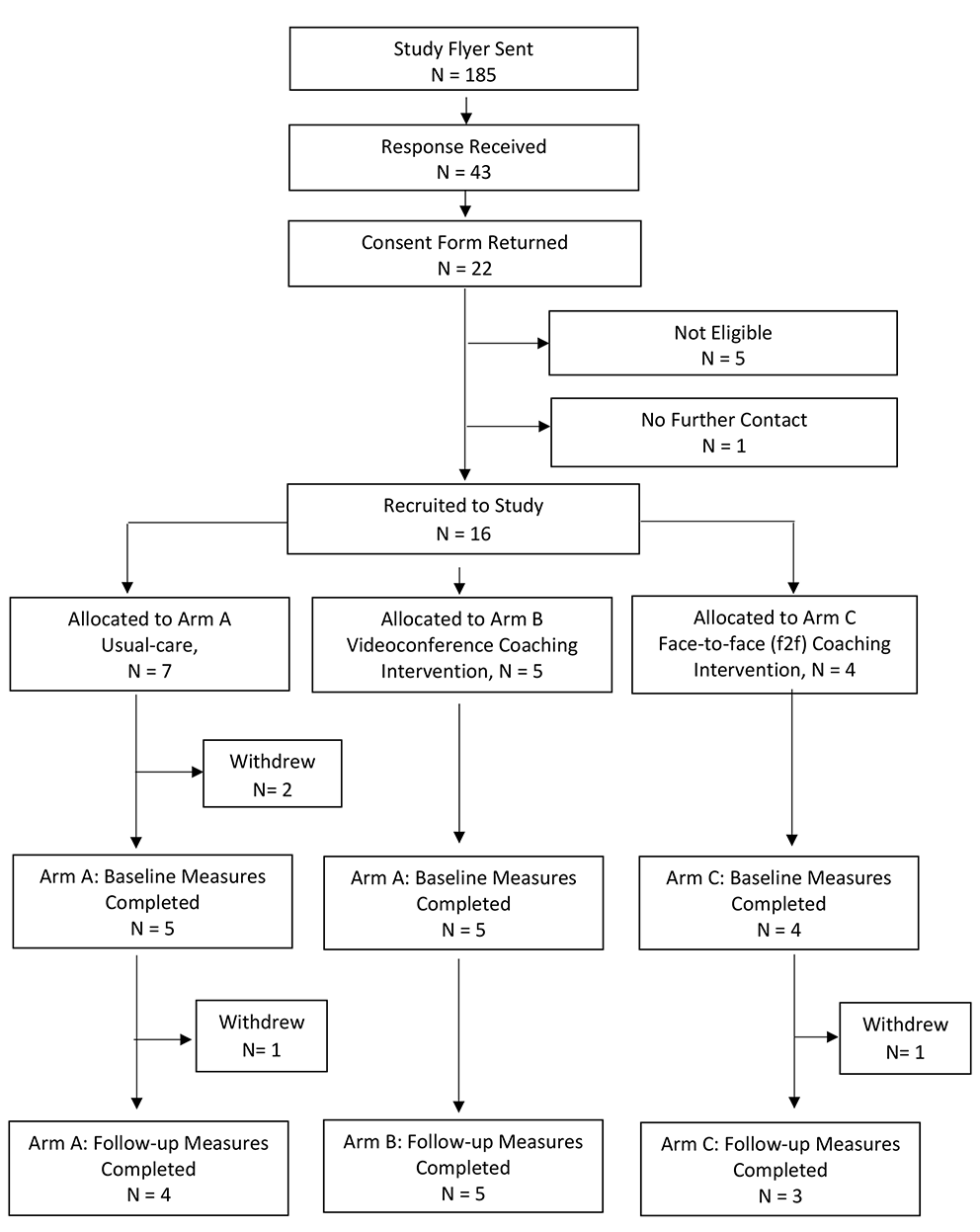
Study Flow Chart

Recruitment, retention and study flow are detailed in Fig. 1.

### Recruitment

128 children at RCH and 57 children at Melton were considered eligible during the recruitment period from December 2018 to May 2019. At RCH, 31 (24%) expressed interest for further information, and 12 (21%) responded with interest at Melton, with a parent information form sent. Of those, 20 families from RCH returned consent forms and 2 from Melton. Three parents reported their child had already accessed an autism assessment elsewhere and were deemed ineligible, and a further child was excluded because their parent was engaged in regular mental health support. One family was lost-to-follow-up before being allocated a study number. Overall 16 families (9% of eligible participants) were recruited. No further recruitment was sought due to researcher time constraints.

The characteristics of recruited families are reported in Table 1.

Of the 16 families, there were 12 mother-child dyads, 1 father-child dyad, and 3 mother-father-child triads (hereon groups will be referred to as participants). Recruited child participants were 11 males and 5 females. All families lived in Victoria, Australia, with 14 of the 16 families (87.5%) from metropolitan Melbourne.

### Retention

In total, four enrolled families withdrew from the study to yield an overall retention of 75%. Three withdrew from usual-care, and one withdrew from f2f coaching. The two families recruited from Melton were not retained.

### Measure Completion

Primary outcome measure completion (detailed in Table 2) was 100% for retained participants. All parents were able to identify between 2 and 4 functional goals pertaining to their child and family for observation over the study period. Secondary measures were completed by at least 75% of participants. Reasons for reduced completion were the child being too young for age range of questionnaire (n = 2), interpreter not available (n = 2) and questionnaire burden/time limitations (n = 3).

Most participants (n = 10, 92%) attended two baseline sessions to complete study measures. Baseline sessions occurred within 2 weeks of each other for all participants. The mean duration between final baseline session (T_0_) and follow up (T_1_) for all families was 5.1 months (SD of 1.0 months). There were no significant differences between study arms for duration between baseline and follow up (F(2, 9) = 1.39, p = 0.299). All participants, apart from those that used interpreters (n = 2), completed follow-up measures within one hour.

### Acceptability of Intervention from Parent Report

Participants who received coaching intervention were asked about aspects of the intervention and its practicality via an optional, anonymous questionnaire which asked about intervention attributes such as location, duration, and satisfaction with outcomes (included as supplementary material). All questionnaire responders (N = 6, 75%) reported that they agreed or strongly agreed that they were satisfied with the intervention overall.

### Implementation, Integration and Practicality

#### Intervention Implementation

All retained participants in the intervention arms participated in 4 interventions sessions, lasting between 35 min and 1 h and 10 min. Interval times between sessions ranged from 1 week to 5 weeks (median of 2 weeks). Strategies to address goals identified at baseline were developed and discussed throughout the sessions in line with OPC principles. Participants were able to trial strategies developed during coaching across relevant contexts and reflect on those in subsequent sessions. All participants in Videoconference Coaching were all able to download and use videoconference technologies to engage in the intervention.

#### Implementation and Practicality of Care (Measure of Processes of Care − 20)

At baseline, 8/12 (75%) of participants were accessing services of an ongoing nature to enable ratings of service quality across five subtests of the MPOC-20. All participants rated care processes at follow-up. For those with available pre and post data, gains occurred with higher frequency for those in the intervention arms (n = 4) across the five subtests of the MPOC-20 (n = 13/20, 65%), than in the usual-care (n = 4) group (n = 8/20, 40%). Greatest gains in the intervention arms were seen for the subtests of Providing General Information (mean change of 4.4 versus 1.0 for usual-care), Providing Specific Information (mean change of 2.8 compared with 0.8 for usual-care) and Coordinated Care (mean change of 2.5 compared with 0.3 for usual-care).

#### Parent Report

Participants who completed the post-hoc feasibility questionnaire agreed that coaching sessions were easy to take part in, and the number of sessions were appropriate for family needs. Responders who participated via videoconference agreed that technologies were easy to use and download, and that the location of intervention (their homes) was a good fit. Two of the 3 families (66%) in f2f coaching were uncertain whether the location was a good fit. Most who received intervention (83%) found the length of the sessions to be appropriate.

#### Cost, Resources, Time

Participants required a mean of 2.5 h of clinician researcher time to complete baseline and follow-up measures. In the intervention arms, participants required a mean of 4 h for the four coaching sessions. Those allocated to f2f coaching had additional travel time to and from sessions, varying from 50 min to 3 h of travel time, and parking costs at approximately $8.00 per hour. Participant’s funded their own internet access and devices for Arm B (intervention via videoconference).

### Adaptation and Expansion

OPC was successfully delivered in a 4-session format for participating families, with adequate fidelity in both videoconference and f2f modalities. Four participants were randomly selected for OPC fidelity measurement, from groups of 3 enrolled participants, in line with recommended procedures. Fidelity scores ranged from 72 to 84%, with a mean score of 81% (Graham et al., 2020).

From the post-intervention questionnaire, most participants (n = 5, 83%) reported that they would recommend the intervention to others who are either waiting for an autism assessment, or to families of children with additional support needs.

### Limited Efficacy Testing (Primary Outcomes)

In the intervention arms, 6 out of 8 participants (75%) moved on average 2 points or greater on the performance and satisfaction scales. In the usual-care group, 1of 4 (25%) participants moved 2 points or more on average on the performance and satisfaction scales. Table 2 details mean change scores and effect size calculations for performance and satisfaction ratings from T_0_ to T_1_ across each intervention arm.

**Table 2:** Goal Attainment Scores – Canadian Occupational Performance Measure (COPM) 6th Edition

Study Arm	Mean (SD) Pre	Mean (SD) Post	Mean Change (SD)	Effect Size η_p_^2^
**Performance**				
Arm A: Usual-care	2.2 (0.8)	3.5 (1.3)	1.3 (1.8)	13.302
Arm B: Videoconference coaching	3.0 (1.2)	6.1 (1.3)	3.1(1.4)	
Arm C: Face-to-face coaching	2.4 (0.6)	5.6 (1.7)	3.2(1.8)	
**Satisfaction**				
Arm A: Usual-care	2.5 (1.4)	4.3 (1.6)	1.8 (1.7)	8.824
Arm B: Videoconference coaching	2.7 (1.7)	6.6 (1.1)	3.9 (1.9)	
Arm C: Face-to-face coaching	1.4 (0.7)	5.4 (2.4)	4.0 (2.0)	

### Limited Efficacy Testing (Secondary Outcomes)

Standardised and non-standardised measures were collected to inform limited efficacy findings and explore participation rates for tool and data completion. Table 3 details completion rates and findings for secondary measures.

**Table 3:** Secondary Measure Findings

Outcome Measure (OC)	Completion Rate	Arm A Usual-Care			Arm B Videoconference Coaching			Arm C Face-to-face Coaching		
		T_0_^a^	T_1_^b^	Change	T_0_	T_1_	Change	T_0_	T_1_	Change
Current Service Access: Mean (SD) of numbers of community services accessed^c^	12 (100%)	1.8 (1.7)	3 (2.1)	1 (0.8)	1.6 (1.5)	3.2 (1.3)	1.6 (0.5)	1.3 (0.6)	4 (1)	2.7 (0.6)
Parenting Stress Index (PSI) Short Form – % ile Score (SD)^d^	11 (96%)	73.3 (32.9)	80.1 (31.2)	7.3 (9.5)	54.0 (21.5)	36.0 (30.5)	-14.5 (21.4)	61.7 (49.1)	66.3 (38.4)	4.7 (11.7)
Beech Family Quality of Life (FQOL) – Range Minimum to Maximum subtest scores on 1–5 scale^c^	9 (75%)	3.0–5.0	2.5–4.75	-0.5 – -0.25	2.5–5.0	3.0–5.0	0.5–0.0	1.0–5.0	2.0–5.0	0.0 – 1.0
Vinelands Adaptive Behaviour Scale (VABS) – Composite Score: Group Mean (SD)^c^	11 (96%)	59.7 (19.9)	64.0 (11.1)	4.3 (8.6)	71.2 (14.9)	75.0 7.5)	3.8 (7.8)	57.3 (10.0)	58.0 (5.6)	0.7 (4.5)
Social Responsiveness Scale (SRS 2) – Total T Score: Group Mean (SD)^d^	9 (75%)	71 (22.6)	82 (9.9)	11 (12.7)	64.8 (15.7)	63.2 (13.5)	-1.6 (4.0)	86 (2.8)	85.5 (6.4)	-0.5 (9.2)

^a^T_0_ = Baseline result

^b^T_1_ = Follow up result

^c^ = higher number reflects a positive change

^d^ = lower number reflects a positive change

## Discussion

This pilot and feasibility trial explored the use of pre-assessment Occupational Performance Coaching or OPC, in addition to service navigation support, to better utilise waiting times that may in turn improve family and child outcomes. It was the first to compare usual-care, face-to-face and videoconference coaching for families of children waiting for an autism assessment. Findings indicate that both the study and the intervention were feasible for participants, with retention meeting pre-designated standards, and high ratings of intervention acceptability and practicality from parent reports. Parent-reported satisfaction with OPC, when applied to children and families awaiting autism assessment, is in line with previous OPC studies (Graham et al., 2013, 2020).

Retention was higher in the intervention arms of the study, and highest for videoconference coaching, with no withdrawals. These findings support growing evidence that videoconference-delivered coaching is feasible and acceptable to families, in line with shifts in recent years to this mode of intervention delivery (Eapen et al., 2021; Gentry, Puspitasari, McKean, Williams, Breitinger, Geske, Clark, Moore, Frye, & Hilty, 2021; Taylor, Caffery, Gesesew, King, Bassal, Ford, Kealey, Maeder, McGuirk, Parkes, & Ward, 2021). OPC is built on principles of connection and sharing, and emphasises explicit relationship development with autonomy support strategies (Graham et al., 2020). These attributes are likely to be facilitative in engaging participants in the intervention from early in sessions, with no barriers observed in the videoconference modality in relationship-building or support provision. The foundations of OPC and known benefits of goal-setting (Cusick et al., 2007) are likely contributors in observed retention in this study, which was similar to other feasibility studies exploring occupation-focused coaching (Kessler et al., 2017; Little et al., 2018). .

All participants made gains on selected goal performance and satisfaction, measured by the COPM. This is not unexpected given enrolled participants were likely motivated to seek services and support for their child and family within and outside of the study. Of note was the greater magnitude of change in goal performance and satisfaction in the intervention arms, compared with usual-care. These preliminary efficacy findings are important because they support the notion that OPC can help families achieve functional goals for the child and family, prior to receiving information provided following diagnostic assessment. This is a critical time to act when considering lengthy waitlists and optimal time for neurodevelopmental change, and findings are in contrast to the notion that diagnostic clarification is required before individualised therapy can commence (Mandell et al., 2010; Matson, 2007). This finding, in combination with high intervention fidelity ratings, provides support for further efficacy testing in an appropriately-powered RCT, to explore potential intervention benefits for families on a larger scale.

The variation in access to existing services by study participants, both at baseline and across the study period, is likely to reflect both variable clinical presentations and service access inequities. At baseline, some families were not yet accessing any intervention services, whilst others had engaged multiple therapies prior to study enrolment. Participants’ service access also changed over the study period, with slightly higher mean increases observed in the intervention groups for numbers of services accessed. These findings provide further support for continued exploration of coaching while waiting for autism assessment, which may be able to facilitate more equitable access to needed services in this period. From a study viewpoint, service access variability is likely to interact with efficacy outcome measures and subsequent RCT findings. A future RCT will need to consider closer tracking of the number, type, duration, and intensity of current and newly-engaged services across the study period, to allow for further analyses relating to their contribution.

Secondary measures were feasible for most families to complete at baseline and follow-up. Results pertaining to secondary measures were not powered to assess efficacy, especially given the variable nature of child developmental trajectories and variation in service engagement observed in the sample. Initial positive trends for decreased parental stress, particularly in the videoconference arm of the study, are nonetheless a promising preliminary finding. Parental stress relating to caring for children diagnosed with ASD, and those waiting on waiting lists, is elevated compared to those not waiting or diagnosed (Batool & Khurshid, 2015; Feinberg et al., 2016; Rivard et al., 2014). It is important to further evaluate the efficacy of pre-diagnostic coaching, such as OPC, in reducing parental stress, particularly when delivered via videoconference. Data on parent sense-of-competence was not collected in this study, and should be included in a future trial, in line with recent recommendations for coaching studies involving primary caregivers (20).

The sample size for this study was small due to study design, recruitment limitations and study withdrawals, particularly from usual-care. Recruitment may have been limited by the exploratory nature of this study, and it is anticipated that a future RCT imbedded in clinical care will overcome this limitation. Participants receiving usual-care were asked to complete a number of measures without any option for future intervention, which may account for this study arm having highest rates of withdrawal. These limitations can be addressed in future studies by ensuring particular elements, such as standardised best-care assessment and goal-setting, are embedded in standards of care prior to commencement. Recruitment and retention in the field of childhood disability research is an ongoing challenge for investigators, particularly for intervention studies that require time investment from carers of young children (Phoenix et al., 2020). This is despite efforts, such as those undertaken in this study, to ensure participation is cost neutral and burdens are ethically appropriate. The burdens of travel were, and should continue to be minimised by utilising videoconference modalities. Extensive questionnaire completion was noted as a reason for withdrawals in this study, and should also be minimised, with information collection in a future RCT restricted to those of high value in service development decision making. Weighted randomisation may be an additional consideration so that predicted withdrawals can occur without compromising RCT aims and minimum sample size requirements. In this trial randomisation was blinded, however, the researcher completing data collection was not blinded to arm allocation. To avoid bias, a future RCT should be double-blinded.

Interpreters were used for two primary-carer participants who were not proficient in English. Both required the maximum permissible time of two one-hour-long sessions to complete baseline measures, and a small number of secondary measures were not completed. The optional post-intervention questionnaire was not completed by these caregivers, and the applicability of feasibility findings to individuals with English as a second language should be interpreted with caution. Neither family withdrew from the study, despite time requirements and communication challenges. Extended time provisions, in addition to use of questionnaires that have been validated in a participants’ first language, would need to be considered in future RCT planning to continue to ensure equitable family inclusion.

Findings indicate that the present study was able to be implemented as designed, with high retention rates and parent-reported satisfaction, as well as preliminary findings indicating the potential to improve child and family outcomes prior to autism assessment. Of particular note are the positive findings around feasibility of OPC when delivered via videoconference, and the positive outcome trends observed in this intervention arm. Given the pandemic-related service changes currently occurring worldwide, and growing support for videoconference interventions broadly, it is prudent to focus on further efficacy testing of OPC and service navigation support delivered using this modality. There is the potential to turn wasted waiting times into active intervention periods that can benefit the child, parents, and family.

## Declarations

### Trial Registration

ACTRN12620000164998
